# Bee Venom: An Updating Review of Its Bioactive Molecules and Its Health Applications

**DOI:** 10.3390/nu12113360

**Published:** 2020-10-31

**Authors:** Maria Carpena, Bernabe Nuñez-Estevez, Anton Soria-Lopez, Jesus Simal-Gandara

**Affiliations:** Nutrition and Bromatology Group, Analytical and Food Chemistry Department, Faculty of Food Science and Technology, University of Vigo, Ourense Campus, E-32004 Ourense, Spain; maria.carpena.rodriguez@uvigo.es (M.C.); bernabenunez16@gmail.com (B.N.-E.); antonsoria@hotmail.es (A.S.-L.)

**Keywords:** bee venom, biological properties, health applications, peptides, enzymes

## Abstract

Bee venom (BV) is usually associated with pain since, when humans are stung by bees, local inflammation and even an allergic reaction can be produced. BV has been traditionally used in ancient medicine and in acupuncture. It consists of a mixture of substances, principally of proteins and peptides, including enzymes as well as other types of molecules in a very low concentration. Melittin and phospholipase A2 (PLA2) are the most abundant and studied compounds of BV. Literature of the main biological activities exerted by BV shows that most studies focuses on the comprehension and test of anti-inflammatory effects and its mechanisms of action. Other properties such as antioxidant, antimicrobial, neuroprotective or antitumor effects have also been assessed, both in vitro and in vivo. Moreover, human trials are necessary to confirm those clinical applications. However, notwithstanding the therapeutic potential of BV, there are certain problems regarding its safety and the possible appearance of adverse effects. On this perspective, new approaches have been developed to avoid these complications. This manuscript is aimed at reviewing the actual knowledge on BV components and its associated biological activities as well as the latest advances on this subject.

## 1. Introduction

### 1.1. Historical Context

Bee venom (BV) has been a study subject since the late 19th century for the potential therapeutic uses of the biomolecules that compound it. This substance is secreted by a gland located in the abdominal cavity of the bees (*Apis mellifera* L.) [1]. A simple fact that illustrates the potential use of the BV in different treatments is related to beekeepers, that are often stung by the bees and unusually suffer diseases like arthritis and other muscles and joints related problems, showing a connection between these two events [2,3]. Since the 3000 BCE, the oriental traditional medicine uses the BV to combat inflammatory diseases [3]. In Europe, Hippocrates (ca. 460-370 BC) was the first to use the term apitherapy and use BV as a treatment of baldness. In the 15th century, Ivan the Terrible was one of the first in use BV to treat other illnesses like gout [4]. Therefore, BV has been historically linked to the therapeutic treatment of anti-inflammatory disorders, skin diseases and rheumatism but has also shown application on treating neurological diseases, asthma, arthritis or infectious diseases such as malaria [1,3].

**Figure 1:**
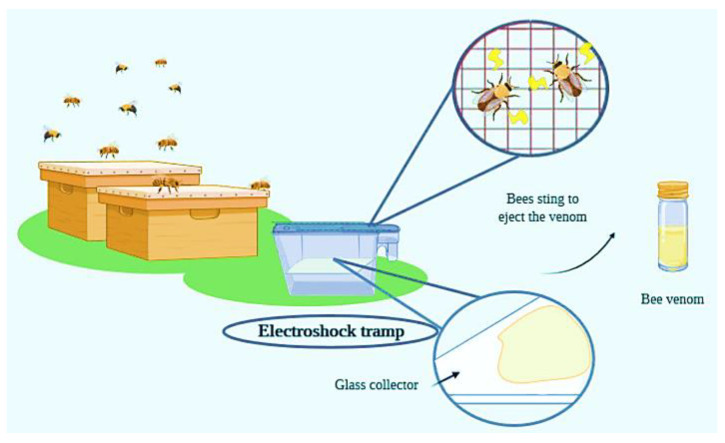
Scheme and process flow of typical bee venom collection by means of using an electroshock trap. Bees are submitted to an electric current, and consequently they eject the venom, which is collected on a glass plate and then transferred to bottles for further processing.

The collection of a considerable amount of BV is hard because a single bee contains a very little amount of venom and to extract it, the bee must sting. To solve this problem, Markovic and Molnar, in 1954, used electroshocks and squeezed to induce the sting of the bees [5]. The efficacy of the method motivated the development of traps that induce the bee sting to collect the venom with the minimum impact on the health of the hive and bees [6]. Modern techniques and traps are derived from the previous one described by [5]. The intensity of the shock, the material that the bees will sting, the duration of the shock and the periods between shocks are so important for the efficacy of the process [7]. In terms of laboratory experiments, BV is extracted by reservoir disruption and/or manual milking, whereas, when more BV is needed, electroshock traps are used. In Figure 1, there is a representative illustration of the process of the BV collection. 

The traditional way of administration of BV is by direct application that is, being stung by live bees. Alternatively, the bee sting can be also simulated by an indirect application with acupuncture needles or directly with an injection of BV in the inflamed area [1,4]. On the other hand, other ways of administration have been used, such as inhalation, iontophoresis or by ointments, as it is easy to apply [4]. Nowadays, the use of BV for the treatment of pathological conditions is accepted and its main components are known; therefore, these facts promote the study of possible therapeutic uses, both by in vitro and in vivo studies [3].

### 1.2. Composition

Principally, most of the insect venoms that sting humans are composed of peptides, proteins, enzymes and other smaller molecules [8]. BV is also composed by these molecules, but its composition is more complex. This complex mixture is based on amino acids (aa), peptides, proteins, enzymes, sugars, biogenic amines, volatile compounds, phospholipids and pheromones, all of them represented in Table 1 and Table 2. Moreover, it is highly aqueous since more than 80% of BV is water [1,9]. Within all these composites, at least, there are more than 18 pharmacologically active compounds.

The two more abundant components of the BV are melittin and phospholipase A2 (PLA2) [1]. Melittin is a peptide that represents the 50-60% of the dry weight (DW) [9,10], the being substance more abundant in BV. Moreover, melittin is the molecule with more different reported biological activities with significant clinical and therapeutic effects; nevertheless, it is the most toxic compound of the BV [11]. The second substance more abundant is the enzyme PLA2, which accounts for around 10–12% of BV; furthermore, it is the second compound with more biological activities [1]. Nonetheless, this enzyme is the most allergenic factor, producing allergenic sensitization in 57-97% of the allergic patients [12].

Other components that have minor presence in BV but exhibit significant biologic activities are peptides such as apamin, mast cell-degranulation peptide (MCD), secapin, adolapin or enzymes such as the hyaluronidase. Apamin is a neurotoxic peptide of 18 aa that has the capacity of blocking the Ca^2+^-activated K^+^ channel [13]. MCD contains 22 aa and it is about 1-3% of BV. Moreover, it has a strong anti-inflammatory activity [14]. Secapin exhibits three attractive biological activities: anti-fibrinolytic, anti-elastolytic and antimicrobial [15]. Adolapin is a polypeptide that possesses anti-inflammatory activity and analgesic effects [16]. At last, hyaluronidase is considered a spreading factor that helps other BV factors to penetrate the cell [17].

**Table 1:** Major components of bee venom (BV) related to their abundance, biological activity and type of study carried out.

Compound	Molecular Group	% in Dry Venom	Biological Activity	Type of Study	Reference
Melittin and isoforms	Peptide	50–60	-Antibacterial -Anti-inflammatory -Anti-arrhythmic -Anti-secretory -Anti-cancer -Anti-arthritis -Anti-atherosclerotic -Antiviral -Pro-apoptotic -Anti-apoptotic -Analgesic -Anti-fibrotic -Anti-diabetic -Haemolysis -Antiangiogenic -Wound healing -Antifungal -Anti-nociceptive	-In vitro -In vivo -In vivo -In vivo -In vitro -In vitro -In vivo -In vitro -In vitro -In vitro -In vivo -In vitro -In vivo -In vitro -In vitro -In vitro -In vitro -In vivo	[18] [19] [20] [19] [21] [22] [23] [24] [25] [26] [27] [28] [29] [30] [31] [32] [33] [27]
Apamin	Peptide	1–3	-Antifungal -Anti-fibrotic -Anti-cancer -Anti-inflammatory -Anti-atherosclerotic -Antibacterial -Neuroprotection	-In vitro -In vivo -In vitro -In vivo -In vivo -In vitro -In vivo	[34] [35] [36] [37] [38] [39] [40]
MCD	Peptide	1–3	-Anti-inflammatory -Anti-allergic	-In vivo -In vitro	[14] [41]
Secapin	Peptide	1–2	-Antifungal -Antibacterial -Anti-elastolytic -Anti-fibrinolytic	-In vitro -In vitro -In vitro -In vitro	[15] [15] [15] [15]
Adolapin	Peptide	0.1–0.8	-Anti-inflammatory -Anti-nociceptive -Antipyretic	-In vitro -In vitro	[16] [16] [42]
PLA2 (Api m 1)	Enzyme	10–12	-Antibacterial -Anti-arthritis -Antiparasitic -Neuroprotective -Anti-cancer -Antiviral -Inflammatory -Antigenicity -Allergenicity -Nociceptive -Neuronal activation -Nerve regeneration	-In vitro -In vivo -In vitro -In vivo -In vitro -In vitro -In vivo -In vivo -In vivo -In vivo -In vivo -In vivo	[39] [43] [44,45] [46] [47] [48] [49] [50] [51] [50] [52] [53]
Hyaluronidase (Api m 2)	Enzyme	1.5–2	-Spreading factor by hyaluronic acid activation -Allergenicity	-	[54] [55]

**Table 2:** Minor components of BV and percentage in dry weight. Based on [1,8,56,57,58,59].

Compound	Molecular Group	% in Dry
Aminobutyric acid	Biologic amine	1
Dopamine	Biologic amine	0.1–1
Histamine	Biologic amine	0.5–2
Noradrealine	Biologic amine	0.1–0.5
Acid phosphatase	Enzyme	1
Phosphatase	Enzyme	1
PLA B	Enzyme	1
α-Glucosidase	Enzyme	0.6
Acetylcholine	Ester	–
Icarapin	Glycoprotein	–
P, Ca and Mg	Minerals	3–4
Apamin	Peptide	1–3
Cardiopep	Peptide	0.7
Cecropin A	Peptide	–
Melittin-F	Peptide	0.01
Melittin-S	Peptide	1–2
Minimine	Peptide	2–3
Pamine	Peptide	2
Procamine A,B	Peptide	1–2
Secapin	Peptide	1–2
Tertiapin	Peptide	0.1
Phospholipids	Phospholipids	1–3
α-D-Glucosidase	Protein	<1
Dipeptidyl peptidase IV	Protein	<1
Lysiphosppholipase	Protein	<1
MRJP8	Protein	–
MRJP9	Protein	–
Phospholipase B	Protein	<1
Vitellogenin	Protein	–
Glucose, fructose	Sugars	2–4
Complex ethers	Volatile compounds	4–8

### 1.3. Biological Activities of Bee Venom 

BV is a toxic substance that causes some problems related to allergic reactions; however, a big dose is necessary to be lethal. The median lethal dose for an adult is 2.8 mg of venom per kg of body weight. Therefore, for a person of 70 kg, 196 mg of venom is needed to be a lethal dose. Nevertheless, this amount of venom is enormous because in one single bee there is only 0.15–0.30 mg of venom. Hence, as the number of stings necessary to be lethal is around 1300, BV has a reduced risk in terms of therapeutic uses [8,17].

The BV components and the synergy that exists between them could be a door to treat some diseases that nowadays are targeted for modern medicine. BV has diverse biological activities, key in the search for therapeutic effects and possible applications. Some of them were studied by Sobral et al. in 2016, reaffirming the antioxidant, anti-inflammatory and cytotoxic activity of BV [60]. Moreover, BV presents other biological activities such as anti-microbial [15], anti-apoptotic or anti-secretory activity [19]. One of the most attractive compounds in BV is the aforementioned melittin, which will be described later, and that exhibits anticancer effects [61] and enhances muscle regeneration [62]. Studies of the therapeutic effects of BV in diseases like rheumatoid arthritis (RA), Parkinson’s disease (PD), multiple sclerosis (MS) and liver fibrosis (LF) have already been performed [3] and reflect a hopeful future for their treatments. 

Regarding all this information, a compilation of studies of the BV components, their characteristics, their biological activities and the interaction of the BV with biological cells and leaving beings is needed to understand its potential use in modern medicine. For this purpose, the present article is aimed to review the current knowledge on BV as well as highlighting the future perspectives for its use and benefits for human health. 

## 2. Bee Venom Composition

BV is a complex mixture of different types of molecules (Table 1 and Table 2). Even though some of its components were previously mentioned, BV has more than 102 proteins and peptides in its composition [56]. Plenty of researchers have investigated the composition of BV; thus, this section will describe its most important bioactive molecules.

### 2.1. Peptides

#### 2.1.1. Melittin

Melittin, also called Api m4 for its allergenicity, is a cationic, linear α-helical polypeptide formed by 26 aa residues. It is soluble in water, amphipathic and has a molecular weight of 2846.5 Da. Its sequence of aa is GIGAVLKVLTTGLPALISWIKRKRQQ, its chemical formula is C_131_H_229_N_39_O_31_ and it has a hydrophobic N-terminus and a hydrophilic C-terminus [63]. Moreover, several isoforms of melittin have been reported and residues fragments have been synthetized, showing an improvement of certain biological properties, such as antimicrobial activity [1,64].

**Figure 2:**
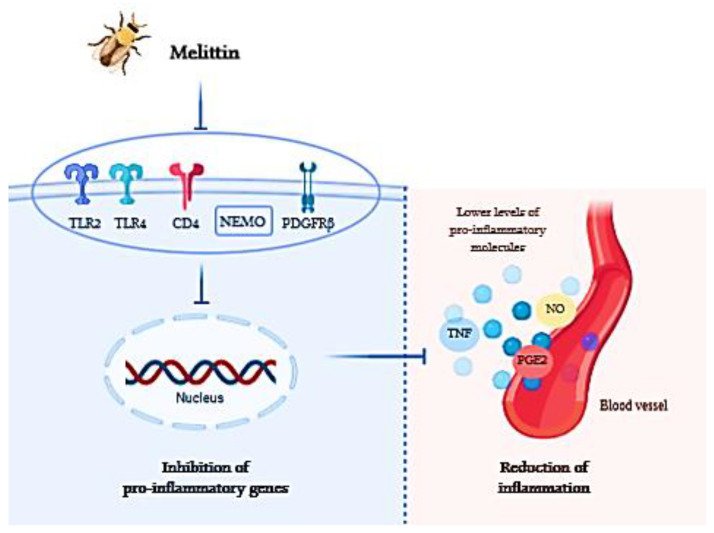
Action mechanism of anti-inflammatory effects of melittin. Melittin inhibits the routes of TLR2, TLR4, CD4, NEMO and PDGFRβ and therefore, inhibits the action of pro-inflammatory genes. This process results in lower levels of pro-inflammatory molecules and the reduction of inflammation. Abbreviations: TLR2, Toll-Like Receptors 2; CD4, Cluster of Differentiation 4; NEMO, nuclear factor kappa-β essential modulator; PDGFRβ, platelet-derived growth factor receptor β;

Furthermore, as melittin is the compound most studied of BV, a series of biological properties have been studied. The anti-inflammatory activity of melittin is produced by various mechanisms. Principally, this mechanism consists in blocking the toll-like receptors (TLR) 2 and 4, the cluster of differentiation 14 (CD14), and the platelet-derived growth factor receptor beta. Moreover, melittin has an inhibitory effect of the nuclear factor kappa-B (NF-kB) essential modulator. All these pathways have the final effect of liberating, to the extracellular medium or the blood vessels pro-inflammatory molecules like inflammatory cytokines, tumor necrosis factors (TNF), nitric oxide (NO) or prostaglandin E2 (PGE2). All these molecules produce inflammatory effects on tissues; therefore, the capacity of melittin to cause the inhibition of the production of these molecules proves its anti-inflammatory effect [65]. At this sense, a scheme of how melittin acts on the inflammation process is shown in Figure 2.

One of the most important characteristics of melittin is its nonspecific cytolytic activity, which comes from the capacity of melittin to conform pores in biological membranes. Melittin is attracted to the anion lipid membranes by its hydrophobic section and its positive charge [66]. Then, melittin inserts itself in the lipid membrane through hydrophobic interactions. This insertion produces vigorous membrane fluctuations. Subsequently, this creates deformed regions where melittin pulls out some phospholipids and replaces their position. At this moment, an asymmetry between the two layers is created, changing the membrane pressure and reducing the energy needed for the insertion of melittin. As a consequence, transient pores will be produced in the membrane by the aggregation of melittin [67]. Moreover, the combination of a big number of pores can collapse the phospholipid bilayer producing the lysis of the cell [68,69]. This interaction with cell membranes gives to this singular peptide the capacity to perform some interesting biological activities, such as anticancer, antimicrobial, antifungal and hemolytic activity, all mentioned in Table 1.

Even though anti-inflammatory properties are probably the most studied on melittin, other capacities have been assessed. For instance, melittin has the potential possibility of being an alternative way to combat virus infections. A complete revision was recently carried out on this subject, summarizing in vitro and in vivo studies and suggesting that one of the main mechanisms of anti-viral activity would be the interaction between melittin and viral envelopes or capsid proteins and thus their interaction with cells [24]. In this respect, melittin associated with nanoparticles has demonstrated the ability to inhibit infectivity of HIV-1NLHX and HIV-1 NLYU2 viral strains and deactivate the viral package [70]. Another mechanism that explains this activity is the interaction of melittin, not only with the virus surface but also with the target of the virus, this is, the host cells avoiding the infectivity. Melittin can inhibit the viral replication by the stimulation of interferon type I (I-IFN); therefore, it could be an excellent pre-treatment method [71]. Uddin et al. in 2016 also found that melittin reduces the mRNA expression in non-enveloped RNA virus; furthermore, it was observed that melittin reduces the amount of virus required to produce cytopathic effect in 50% of inoculated cells [71].

Melittin has the capacity to interact with so many types of cancer cells, some of them related with the final apoptosis. This activity has been evaluated on different cell lines. For instance, melittin can inhibit cell growth of human ovarian cancer cells increasing the expression of death receptors (DR3, DR4 and DR6) and the inactivation of the signal transducers and activators of the transcription 3 (STAT3) pathway, ending in the apoptosis of the cells [72]. Besides, melittin can induce apoptosis in gastric cancer cells through the mitochondria pathways and its concomitant increased generation of free radicals [25]. Melittin can also induce caspase-dependent apoptosis in melanoma cells also by displaying a downregulation of phosphoinositide 3-kinase (PI3K), protein kinase B (AKT), mammalian Target of Rapamycin (mTOR) and 5’ adenosine monophosphate-activated protein kinase (MAPK) signaling pathways [21]. Moreover, melittin can also affect the growth of human hepatoma cells via HDAC2-mediated PTEN upregulation, AKT inactivation and inhibition of the previous mentioned PI3K/AKT signaling pathway [73]. In addition, melittin showed antiangiogenic effects through decreased vascular endothelial growth factor (VEGF) expression by inhibiting the hypoxia-inducible factor-1α (HIF-1α) protein in human cervical cancer cells [31].

Recently, the capacity of melittin to prevent the apparition of cancer cells was also studied. The major cancer associated mortality is a consequence of the metastasis, so the prevention of the dissemination of these cancer cells will help to reduce the number of patients with cancer. In this sense, carrying systems play a key factor on transporting the bioactive molecules to the exact target cells. Xiang et al. in 2019 studied the in vivo capacity of melittin associated with nanoparticles (α-melittin-NPs) to prevent metastasis in liver sinusoidal endothelial cells (LSECs) by immunologic tolerance of the liver. They demonstrated that α-melittin-NPs target and modulate LSECs. The activation of LSECs results in an immunologic response that inhibits liver metastasis reducing the probability of the appearance of liver cancer [74]. 

#### 2.1.2. Apamin

Apamin is a neurotoxic peptide composed of 18 aa residues cross-linked by two disulphide bonds and a molecular weight of 2111.4 Da. Its sequence of aa is CNCXAPETALCARRCQQH and its two disulfide bonds connect position 1-11 and 3-15 [75]. Despite there are different models for the structure of apamin, its study shows a conformation of an α-helix that displays high stability in different pH values. One interesting characteristic of apamin is the permeability to the blood–brain barrier that gives apamin access to the central nervous system (CNS) [76].

Apamin has the capacity of blocking Ca^2+^-activated K^+^ channels, conferring on it cytotoxic and nociceptive activity in nerves, as it is an allosteric inhibitor [77]. Moreover, apamin can activate the inhibitory muscarinic M2 receptors in motor nerve terminals reducing the neuromuscular transmission [78]. Therefore, this capacity gives apamin the potential to be a part of the treatment of different CNS diseases [79]. 

Besides its effects on CNS, apamin is also known as an anti-inflammatory agent able to cause the inhibition of cyclooxygenase-2 and lower the levels of TNF-α, IL-1 (Interleukin-1), IL-6 and NO [37,80]. In this regard, another study showed that apamin was able to suppress Th2-related chemokines and other pro-inflammatory cytokines at the same time as inhibiting the activation of the NF-κB (Inhibitory effect of the nuclear factor kappa-B) and STAT pathways in human keratinocytes cell line [81]. Moreover, other properties have been reported as apamin (2 μg/mL) added to THP-1-derived macrophages incubated with oxidized low-density lipoprotein (50 μg/mL) acts as a suppressor of lipid deposition and of the expression levels of apoptosis-related proteins of the Bcl-2 family, cytochrome c, caspase-3 apoptotic cascade, poly (ADP-ribose) polymerase (PARP) and, at last, in apoptotic cells—in a dose-dependent manner [82].

#### 2.1.3. Mast Cell-Degranulating Peptide

Mast Cell-Degranulating Peptide (MCD) or peptide 401 is formed by 22 aa residues and has a molecular weight of 2587.2 Da. Its structure of aa is IKCNCKRHVIKPHICRKICGKN. Moreover, it has a similar secondary structure to apamin, it also has two disulfide bridges that, in this case, join the aa 3 with 15 and 5 with 19. At physiological pH, it has a net charge of +8 [83,84].

In a low concentration, less than 0.1 mg/mL, MCD produces mast cell degranulation and concomitant histamine release giving to MCD, immunologic properties and facilitating the response of the BV, being responsible for the reddening, inflammation and located pain at the sting site [76,85]. This process has been hypothesized to occur in different ways. In the presence of immunoglobulin E (IgE), MCD peptide will work as an “allergen” unchaining the degranulation of the mast cell and the release of histamine. In the absence of IgE, it is theorized that some groups of MCD resembles to biding site of IgE and, thus, can bind to the high-affinity IgE receptor (FCεRI) of the mast cell, acting as IgE [86]. Moreover, other authors have pointed out that MCD peptide mediates the process of degranulation by increasing the concentration of free cytoplasmic Ca^2+^ [87].

**Figure 3:**
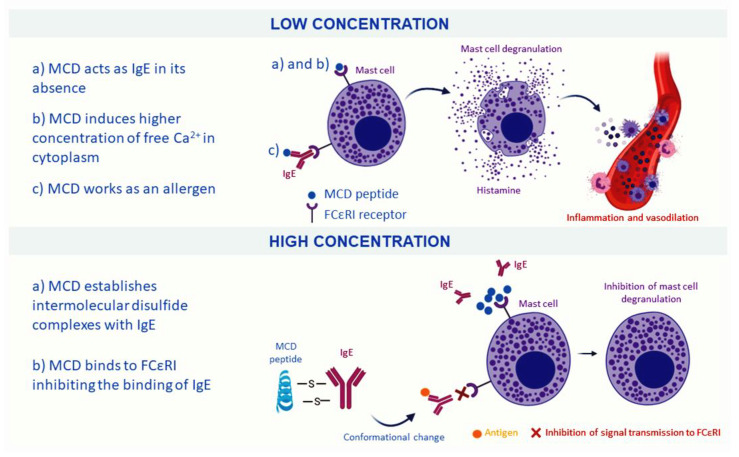
Different effects of Mast Cell-Degranulating (MCD) peptide when it is present in low or high concentration. In a low concentration, MCD induces mast cell degranulation and the release of histamine, resulting in inflammation processes. In a high concentration, MCD inhibits mast cell degranulation and therefore, exerts anti-inflammatory effects.

Alternatively, in high concentrations, MCD acts as an anti-inflammatory compound and inhibits the release of histamine. It has been postulated that MCD peptide and IgE can stablish intermolecular disulfide complexes, causing a conformational change on IgE and inhibiting the signal transmission to the FCεRI receptor. It has also been highlighted that MCD could bind to these receptors inhibiting the binding of IgE, and finally avoiding the histamine release [85,88]. Moreover, MCD can act as a neurotoxin for its capacity of blocking Ca^2+^-activated K^+^ channels producing an increase in neural excitability, as in the case of apamin [57,89]. A scheme of the effects of MCD depending on their concentration is shown in Figure 3.

#### 2.1.4. Minor Peptides

Besides the main peptides of BV (melittin, apamin and MCD peptide), other minor peptides are found in lower quantities. Research on these molecules has been quite scarce, although, as BV peptide, they are expected to show biological effects [15]. Secapin is composed of 25 aa residues with a weight of 2866.5 Da. Its sequence of aa is YIIDVPPRCPPGSKFIKNRCRVIVP with a disulfide bridge in its structure that connects aa 9 with 20 [90,91]. Secapin represents around the 1% of BV composition, although it has been studied that this proportion increases in the case of queen-bee venom glands. However, studies developed to assess the properties of this peptide have been performed with synthetized secapin [92]. It has been reported its potent neurotoxin character and antimicrobial activity [15,93]. However, one of its isomorphs, secapin-1, is a serine protease inhibitor-like peptide that has demonstrated anti-fibrinolytic, anti-elastolytic and anti-microbial activities [15]. Likewise, secapin-2 has shown hyperalgesic and edematogenic effects [94]. Adolapin is a basic polypeptide with 103 amino acid residues. Adolapin shows anti-inflammatory effects as it can block prostaglandin synthesis and inhibit cyclooxygenase activity [16]. Therefore, these capacities give to adolapin the biologicals activities mentioned in Table 1. Furthermore, adolapin can produce an analgesic effect or inhibit lipoxygenase from human platelets [95] and can also interact with PLA2, causing its inhibition [96]. Tertiapin is a peptide formed by 21 aa residues with a molecular weight of 2.460 Da. Its aa sequence is ALCNCNRIIIPHMCWKKCGKK with two disulfide bridges connecting Cys3 with Cys14 and Cys5 with Cys18 [97,98]. Tertiapin interacts with inward rectifier potassium (Kir) channels expressed in epithelial cells, heart and CNS by blocking them. These channels are important in myocytes and are related to sinus node dysfunction (SAD) [99]. Therefore, tertiapin is used as a tool for modulating these channels [89]. Tertiapin has in its aa sequence a methionine that produces oxidation-driven chemical changes. To avoid this issue, the tertiapin-Q was created, a synthetic molecule, where the methionine was changed for glutamine, and which does not produce oxidation-driven chemical changes while conserving all biological activities of the tertiapin [100]. 

At last, other peptides have been reported on BV. Procamines are small peptides of 5 aa residues; principally, there are procamine A and procamine B, both characterized by the presence of histamine in its C-terminal [101]. Minimine is a peptide of 48–52 aa residues and a molecular weight of 6000 Da, the biological activity of which is still unknown [102]. Cardiopep showed beta adrenergic and anti-arrhythmic effects [103]. Apideacin is a proline-rich antibacterial peptide conformed by 18 aa residues that kills bacteria through a bacteriostatic process [104]. However, it should be accounted that the majority of these studies were performed decades ago and no further studies have been developed. 

### 2.2. Enzymes

#### 2.2.1. Phospholipase A2 

Phospholipase A2 (PLA2) or Api m1 is a polypeptide of 134 aa residues with a weight of 15–18 kDa. To maintain its conformational stability, this enzyme has five disulfide bonds that join 9–31, 30–70, 37–63, 61–95 and 105–113 aa residues [105]. There is a wide diversity of PLA2 in nature, so these enzymes have been classified into 16 groups. Particularly, PLA2 derived from bee (bPLA2) belongs to group III. This group of enzymes is calcium dependent and possesses catalytic activity. Moreover, bPLA2 has a highly conserved Ca^2+^ binding loop and a catalytic His/Asp dyad [106].

**Figure 4:**
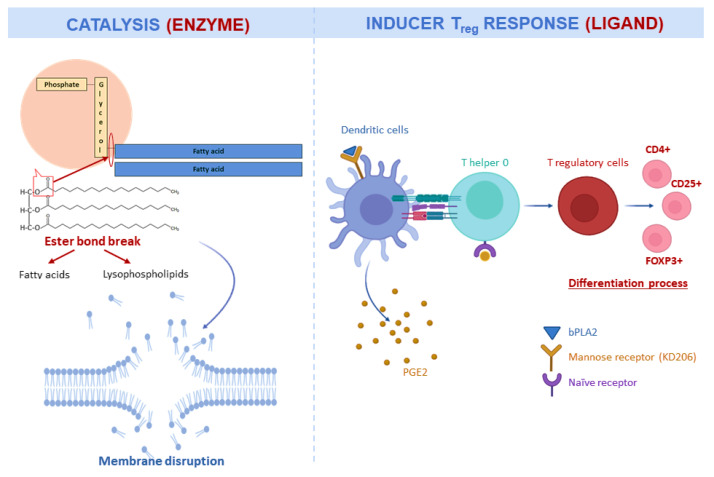
Double behavior of PLA2. This enzyme has the ability of acting as enzyme or as a ligand. As an enzyme, it causes membrane disruption and can exert antimicrobial and cytotoxic effects. As a ligand, it produces cellular signals and modulate immune response.

The principal catalytic activity of bPLA2 is the hydrolysis of the sn-2 fatty acyl ester bond of membrane glycerol-3-phospholipids that results in the liberation of fatty acids and lysophospholipids [107] (Figure 4). This catalytic activity hydrolyses and digests cell membrane components, destabilizing it and making it susceptible to collapse or produce further degradation [108]. PLA2 showed high cytotoxic activity against cancer cells by membrane disruption [107]. The disruption of membranes also gives to bPLA2 antimicrobial activity [109]. Moreover, bPLA2 presents antiviral activity against viruses with lipid bilayers envelopes derived from the endoplasmic reticulum [110].

Furthermore, bPLA2 can also act as a ligand for specific receptors. In this sense, bPLA2 can bind to specific membrane receptors and produce cellular signals independently of their enzymatic activity [111]. There are two types of receptors identified for bPLA2: M-type and N-type. M-type receptors were found in skeletal muscle cells [112], whereas N-type receptors were found in rat brain membrane [113]. N-type receptors are correlated with the neurotoxic activity of bPLA2 [114]. 

bPLA2 is usually considered the major allergen of BV and can upregulate the expression of Foxp3 and thus promote regulatory T (Treg) cell differentiation by the release of PGE2. This ability proposes bPLA2 as an alternative treatment for neuroinflammatory diseases as they mediate anti-inflammatory effects [111,115]. A representation of this regulated expression pathway is shown in Figure 4 [111,116]. This Treg expression has been demonstrated to modulate apoptotic signalling and therefore, it possesses immunomodulatory properties [116]. Meanwhile, bPLA2 can induce a T helper type-2 (Th2) response that is dependent on MyD88 expression in T cells, inducing an immune protective response against future BV exposure [117]. At last, other studies have investigated homologs of bPLA2, such as mammalian PLA2G3, and found that when they are coupled to fibroblast lipocalin-type PGD synthase (L-PGDS), they produce a lipid mediator (in this case, PGD2) that binds to type-1 PGD receptor facilitating mast cell maturation and inducing anaphylaxis [118].

#### 2.2.2. Hyaluronidase

Hyaluronidase or Api m2 is composed of 350 aa residues with a disulfide bridge. The main activity of this protein is to help venom components to penetrate the bloodstream by the degradation of the hyaluronic acid in the extracellular matrix of different tissues [55]. This capacity gives to the protein the common name of “spreading factor”, for helping on the expansion of the venom. Furthermore, hyaluronidase has other activities related to the venom action like the formation of pores, producing membrane disruption and mast cell degranulation [119].

#### 2.2.3. Other Enzymes

Acid phosphatase or Api m3 is a glycoprotein with four sites of glycosylation [120,121]. Their acid phosphatase activity is characterized by their motif RHGXRSP that gives it distinction from the rest of acid phosphatases. Acid phosphatase causes the release of histamine and produces specific IgE that can be used in immunotherapy [120]. Dipeptidyl peptidase IV (DPIV) or Api m5 has a molecule weight of 102 kDa [122]. DPIV has the capacity of cleaves N-terminal Xaa-Pro or Xaa-Ala dipeptides [119] and it is related to the conversion of pro-toxins into their active forms [119,122]. Vitellogenin or Api m12 is the molecule with the highest molecule weight, 200 kDa, in BV [119]. Moreover, it has shown antimicrobial and antioxidant activities [123].

## 3. Biological Activities of Bee Venom

### 3.1. Antioxidant Activity

As previously mentioned, in the BV there are components with antioxidant activity. The efficacy of this activity is usually related to the concentration of melittin, PLA2 and apamin. The antioxidant effects could be caused by the capacity of these compounds to inhibit the lipid peroxidation process and increase superoxidase dismutase activity [60]. However, apart from them, there are other components in BV that have antioxidant activity. For instance, vitellogenin presents antioxidant activity in mammalian cells by the mechanism of direct shielding of the cell against oxidative stress giving to the cells protection against reactive oxygen species [123].

Regarding in vitro experiments, a study assessed the antioxidant activity of bee venom by different methods: 2,2-diphenyl-1-picrylhydrazyl (DPPH) scavenging activity, reducing power, β-carotene bleaching inhibition and thiobarbituric acid reactive substances (TBARS) inhibition. Results showed the antioxidant potential of BV, although it was not linked to any individual compound [60]. Another study evaluated the antioxidant capacity by DPPH, ferric reducing/antioxidant power (FRAP) and 2, 20-azinobis 3-ethylbenzothiazoline-6-sulfonic acid (ABTS) assays, comparing different species of *Apis*. All the venom extracts showed inhibition of DPPH, whereas the highest activity was performed by *Apis dorsata* followed by *A. mellifera* [124].

Recently, the effects of BV on male rabbits were studied. Rabbits were treated with 0.1, 0.2 and 0.3 mg per rabbit by injections under the skin twice a week by a period of 20 weeks [125]. To find out possible changes in the antioxidant activity, during the experiment it was measured the total antioxidant capacity (TAC), glutathione S-transferase (GST), glutathione content (GSH), glutathione peroxidase (GPx), superoxide dismutase (SOD), malondialdehyde (MDA) and TBARS. The results showed an increase in the GST and GSH in the treated rabbits. Moreover, MDA and TBARS levels were lower. These results confirmed the antioxidant activity of BV. Furthermore, BV produced an improvement of the reproductive performance closely related to the improvement in the antioxidant activity of the semen [125]. Adurrahim et al. in 2019 studied the antioxidant activity of BV in rats with RA by measuring the plasma total antioxidant status (TAS), total oxidant status (TOS) and oxidative stress index (OSI). The results showed that those rats with RA treated with BV increased their TAS levels and decreased TOS and OSI levels, not showing any difference between the group treated with high or low doses [126]. Moreover, the antioxidant activity has been demonstrated on induced gastric ulceration in rats. The experiment showed that, compared with those animals treated only with acetylsalicylic acid (ASA), those treated with BV and ASA attenuated lipid peroxidation and antioxidant enzyme activity [19].

### 3.2. Antimicrobial Activity

BV has been investigated for its antimicrobial properties, namely for two of its components, although studies on the whole BV have been developed (Table 3). This antimicrobial activity comes principally from the antimicrobial peptide melittin. The principal mechanism of antimicrobial action of melittin is the capacity to disrupt the biological membranes as it was described in previous sections. On the other hand, PLA2 also presents antimicrobial properties [1,127]. Moreover, other components present antimicrobial activity; for instance, vitellogenin acts as an antimicrobial peptide inducing damages in the cell membranes of bacteria [123]. As it can be seen in Table 3, BV components possess antibacterial activity against Gram + and Gram ‒ bacteria, and, also, antifungal effects have been studied against some species such as those belonging to *Candida* genus [1,127]. In this sense, a recent review article collected the information about the antifungal effects of melittin [33]. Other compounds, such as secapin, have proved antibacterial and antifungal effects [15]. 

BV and, particularly, melittin have demonstrated their capacity to disrupt cell membranes and interact with superficial molecules of cells. This ability could also show potential for using it in antiviral therapy. Studies in animal and plant viruses have already shown the potential antiviral activity of BV [128,129]. BV was evaluated against Green Fluorescent Protein fused Vesicular Stomatitis Virus (VSV-GFP), showing that BV inhibited the replication of the virus by three different treatments: pre-treatment, co-incubation and post-treatment experiments. Moreover, melittin exhibited antiviral effects against enveloped virus by its lytic capacity. Melittin demonstrated antiviral effects against influenza A virus (PR8), vesicular stomatitis virus (VSV), respiratory syncytial virus (RSV) and herpes simplex virus (HSV) [71]. Furthermore, melittin also exhibited antiviral effects against viruses without a viral membrane, such as enterovirus-71 (EV-71) and Coxsackie virus (H3). In the pre-treatments with BV, it was observed that virus replication was lower, which is ligated to a major production of I-IFN. Besides, melittin showed no toxicity in a study in vivo with mice that before treatment with melittin showed resistance to a lethal dose of influenza a virus [71]. At last, it has been also suggested using BV and, particularly, PLA2 as an antiparasitic agent in the treatment against some organisms such as *Trypanosoma brucei brucei* or *Plasmodium falciparum* [44,45].

### 3.3. Anti-Inflammatory Activity

There are at least four main compounds of BV that present anti-inflammatory properties. The anti-inflammatory activity of melittin was tested against acne vulgaris, neuroinflammation, amyotrophic lateral sclerosis, atherosclerosis, arthritis and liver inflammation [65]. 

In relation to inflammatory skin diseases, *Cutibacterium acnes* plays an important role as it induces the activation of TLR2 and TLR4 that produce the liberation of cytokines and chemokines, such as TNF-a, IL-1b, IFN-g and IL-8, inducing the inflammation process. TLRs modulate the activation of NF-kB and MAPK signaling pathways that are involved in inflammatory gene expression. Pathways of NF-kB are conformed by a group of inducible transcription factors that have critical activities in the host immune and inflammatory response [130,131]. On the other hand, MAPK pathways are implicated in the proliferation, survival and differentiation of cells, also related to the inflammation process [130,132]. 

The treatments with melittin modulate TLR pathways activation and inhibit the expression of inflammatory cytokines. In vitro, melittin can suppress the activation of nuclear NF-kB p65 and inhibit the p38 MAPK signal [130]. Therefore, this function produces anti-inflammatory activity by NF-kB signaling and p38 pathway. In vivo, melittin also showed anti-inflammatory properties by the modulation of NF-kB and AP-1 transcription factors [130].

In recent studies, BV demonstrated to have anti-inflammatory activity by a topical route of administration against atopic dermatitis. The anti-inflammatory effect is caused by a reduction of the IgE level, cytokine release and NF-kB and MAP kinase activities. The reduction in NF-kB and MAPK activities generates the inhibition of lipopolysaccharides (LPS)-induced inflammatory responses and the TNF-α/IFN-γ-dependent inflammatory response. Moreover, a reduction in the activity of MAPK affects the regulation of the NF-kB signals that produce changes in the cytokine release and in the expression of genes such as COX-2 and iNOS, both inflammatory genes [133,134]. Therefore, the reduction in the inflammation decreases the skin damage produced by atopic dermatitis [133]. 

RA is one of the most common inflammatory pathologies, whose prevalence is between 0.2 and 0.9% depending on the country [135]. BV has been shown to have medicinal properties against arthritis [136], as in the case of a recent study that showed the effects of BV in rats with induced arthritis. The group that presented the best answer to the treatment was the group treated with 2 mg/kg of BV administrated subcutaneously during 15 days [126]. This dose of BV did not alter the liver and kidney function. This group showed lower levels of inflammatory cytokines, such as IL-1β, IL-6, TNF-α and TGF-β1 than the positive control. This decrease in proinflammatory cytokines could be due to the fact that PLA2 is one of the major inflammatory triggers in RA, and as melittin can conform a melittin-PLA2 complex, it could cause the inhibition of the proinflammatory activity of PLA2 [126,137].

Another important inflammatory disease is gouty arthritis. Gouty appears with the accumulation of monosodium urate crystals in the intra-articular space producing inflammation [37,138]. The intraperitoneal and oral administration of BV and apamin (0.5 and 1 mg/kg) not only showed a decrease in the inflammatory cytokines but also deceased the paw edema and pain in induced gouty mice. This could be a response to the lower inflammation produced by the suppression of NF-kB and NLRP3 inflammasome [37].

### 3.4. Neuroprotective Effects

Neurodegenerative disorders are linked to the neuroinflammation of the chronic activation of glia cells and microglia. Some of the most important neuronal diseases are PD, Alzheimer’s disease (AD) and amyotrophic lateral sclerosis [57]. Some components of BV, such as PLA2 and apamin, have been studied as anti-neuroinflammation agents to improve the efficacy of some drugs against neurodegenerative disorders [40]. The relation between inflammation and neuronal diseases makes that previous section strongly related to the neuroprotective effects of BV. 

In AD, neuroinflammation is critical in the development and pathogenesis of the illness. The upregulation of pro-inflammatory molecules and the microglial activation produce the accumulation of amyloid beta, a peptide related with AD [46]. Due to the insoluble character of amyloid beta, its accumulation conforms an extracellular senile plaque deposit that affects the normal function of the brain producing cognition and memory impairments [139]. Moreover, microglial cells release pro-inflammatory molecules like cytokines TNF, IL-1β and IL-6 [140]. NF-kB also plays an important role in the inflammation of AD. In postmortem brain tissues, it has been found that NF-kB immunoreactivity was increased in astrocytes and microglia. Therefore, the downregulation of NF-kB could help to decrease the inflammation in AD [141].

bPLA2 has shown in previous studies the capacity to increase Treg population, suppress microglial activation and possess protective effects related to anti-inflammatory and anti-immune response [142,143,144]. Therefore, PLA2 was tested against AD in vitro and in vivo. To simulate AD clinical effects, mice were treated by systemic treatment with LPS that stimulate the pro-inflammatory reactions, lead to memory dysfunction, generate amyloid beta and activate astrocytes and microglial cells [46,145]. In mice treated with 0.2 and 2 mg/kg of PLA2 by intraperitoneal injection and LPS stimulated, the results were promising. The administration of PLA2 reduced the expression of amyloidogenic and inflammatory proteins like amyloid precursor protein, COX-2, Bace1 and iNOS that have been previously increased by the treatment with LPS. Moreover, PLA2 inhibited GFAP and IBA-1 expresion and inflammatory cytokines release, both in vivo and in vitro. This inhibition produced a decrease in amyloidogenesis levels and improved memory impairment. Furthermore, PLA2 reduced microglial activation and the generation of amyloid beta by the upregulation of Treg population. As well as melittin [130], PLA2 mediated the inhibition of NF-kB that could help to reduce amyloidogenesis, neuroinflammation and improve memory function [46].

Parkinson’s disease (PD) is one of the most important neurodegenerative illness, since 3 out of 100 people over 65 years old have PD [146]. As well as AD, the inflammatory response by the activated microglia producing pro-inflammatory cytokines also plays a critical role in PD. Moreover, the infiltration of T cells into the brain and glial cell activation are important factors on the clinical development of PC that produce the dead of dopaminergic cells [147]. As was mentioned before, bPLA2 has anti-inflammatory effects and induces protective effects against inflammatory conditions [117,148]. In a recent study, a mixture of 78% of bPLA2 and 15% of melittin was tested against PD in mice [49]. To initiate PD in mice, the 1-methyl-4-phenyl-1,2,3,6-tetrahydropyridine model was used. The results show that bPLA2 reduced the inflammation by the activation of Treg cells. Moreover, bPLA2 activity decreased the levels of dopaminergic cell loss. The results suggest that PLA2 could be a potential drug to increase the dopaminergic cells survival in PD [49].

### 3.5. Antitumor Effects

The search for natural products with antitumor properties has been so intensive during recent years. The central aim of this search has been finding products with inhibitory activity against tumor cell growth and metastasis and able to induce and control apoptosis. Several studies reported that BV and its components present some of these properties, like apoptosis induction and necrosis and growth inhibition of different tumor cells [47,149,150,151,152,153]. The main components in BV that present antitumor effects are melittin and PLA2. Moreover, the interactions between them produce antitumor activity [36]. 

The apoptotic activity against cancer is the most attractive activity to reduce tumor cell growth. Melittin is the component of BV that has higher cytotoxic activity against tumor cells. The first study that demonstrated the anti-cancer effect of melittin showed that the apoptosis of cancer cells was produced by the inhibition of calmodulin in leukemic cells. This inhibition was caused by the pump activity of Ca^2+^ channels producing a big increase in Ca^2+^ concentration that consequently induced cell death [154]. Since that discovery, several studies have been performed with different types tumor cell lines to search for the antitumor effects of melittin and their mechanisms of action.

BV and melittin showed the inhibitory activity of cancer cell growth in prostate cancer. This effect was caused by the down-regulation of antiapoptotic gene products such as Bcl-2, XIAP, iNOS and COX-2 [149,155]. Therefore, this down-regulation produced the inhibition of the transcriptional activity of NF-kB that is related to apoptotic cell death. The inactivation of NF-kB signaling was caused by the impairing IkBα phosphorylation by the inhibition of p50 and p65 translocation [149]. Another pathway of BV and melittin induced apoptosis was studied in ovarian cancer cells. In this study, melittin presented an anticancer effect by the induction of death receptors and inhibition of the JAK2/STAT3 pathway [72]. The main action to decrease the growth of ovarian cancer cells was the inactivation of STAT3 and the overexpression of death receptors DR3, DR4 and DR6. The expression of these DR produced the caspase-8 dependent activation apoptosis [72]. 

Alternatively, apoptosis is not the only pathway used against cancer by melittin. In mice with murine Lewis lung carcinoma in blood, melittin reduced the proliferation of tumor cells without apoptosis. The treatment with melittin decreased the number of tumor-associated macrophages (TAM), especially CD206^+^ M2-like TAMs in tumor stroma. The number of VEGF^+^ and CD31^+^ cells in tumor tissues was reduced because of the decrease in CD206^+^ M2-like TAMs. This fact demonstrates the anti-angiogenic effect of melittin [156]. Other mechanisms that produce cancer cell death is related to the ability of melittin to interact with phospholipidic membranes. This interaction produces pores that can collapse the cell membrane producing the lysis of the cells [67,68]. This capacity was studied against gastric and colorectal cancer. With a dose of 20 µg/mL of melittin, the reaction of the cancer cells in vitro was very quick; melittin only needed a minute to produce granulation, blebbing and cell swelling. Furthermore, 15 min after the initiation of the treatment, there was complete death [157]. However, this lytic effect is not specific to cancer cells and can produce the lysis of other healthy cells. In this sense, one solution is the use of carriers such as nanoparticles to limit the action of melittin in the target cells. In a recent study, the association of melittin with nanographene oxide and melittin with nanodiamonds was studied against breast cancer [158]. This association produced an increase in the toxic effect on cancer cells than melittin alone. Moreover, melittin with nanodiamonds was able to protect healthy cells against the lytic effect of melittin. Furthermore, it was observed that the necrosis level decreased [158].

## 4. Clinical Applications

Considering all the biological activities exerted by BV and the processes where it can act as mediator, it would not be surprising that its use could reach therapeutic purposes. Traditionally, BV has been known for its anti-inflammatory, anti-apoptosis, anti-fibrosis and anti-arthrosclerosis effects, but more recently other approaches have been also highlighted as its action on neurodegenerative and circulatory diseases [3]. 

Firstly, BV can be administrated by different therapy methods: direct sting of the bee, BV injection or BV acupuncture (also called apitherapy). Most of studies and practices uses this last option due to their benefits: BV bioactivity coupled to mechanical stimulation of acupuncture [3,137]. Apitherapy has been traditionally used in medicine, but scientific studies on this discipline are less common. In this regard, and with the purpose of assessing the therapeutic potential of BV, a study performed in humans demonstrated that both acupuncture and BV acupuncture showed efficacy as adjuvants in PD treatment, when adults were stimulated on 10 acupuncture points, twice a week for 8 weeks [159]. Likewise, in 2017, a double-blind random trial assessed the synergistic and enhancement effects of BV and acupuncture [160]. Khalil et al. in 2015 examined the capacity of BV acupuncture against rotenone-induced oxidative stress, neuroinflammation and apoptosis processes in mice [161]. In terms of pain and antinoceptive effects, a study showed that, by the injection of the 0.25 mg/kg of BV, the inflammatory pain threshold related to arthritis was lowered after three weeks [162].

Along the text, examples of in vitro and in vivo experiments have been given, although the performance of in vivo studies is of greater importance and closer to reality. In vivo experiments have been almost always developed in mice, rats or murine models. For instance, recent studies have proved the positive effects of BV on amyotrophic lateral sclerosis [163], chronic prostatitis [164], gouty arthritis [37] or AD [144], among other diseases. Studies have also been developed on rabbits. This is the case of a recent work that investigated the role of apamin on small conductance calcium-activated potassium current and found that it induced more action potential duration in long term cardiac memory, showing up-regulation of those sites [165]. Other models less common have also been searched. For instance, some authors investigated the role of apamin treatment on the equilibrium recovery stabilization in a cat model submitted to unilateral vestibular neurectomization [166]. On the other hand, studies in humans have been also performed, although they are scarcer, and sometimes they show varied and contradictory results. For instance, a study developed in 2012 reported an improvement by the application of BV acupuncture [159], whereas more recently, an experiment with BV injection once a month over 11 months showed no significant effect when compared to placebo [167]. Moreover, a review reported that while the inhibition of inflammation and nociceptive behavior occurs in rodent models, further study is necessary in humans, with better designed trials and larger sample numbers [58]. 

However, notwithstanding the latent therapeutic potential of BV, there is a parallel reality of potential side effects or allergic reactions linked to BV composition. This aspect is one of the targets for the development of safe practices [168]. A systematic review provided a summary of the studies developed in apitherapy and their related adverse effects. The manuscript reviewed a total of 145 studies and found that 28.87% of BV-therapy-treated patients experienced adverse effects, accounting for an increase in relative risk of 261% when compared to normal saline injection. This study also highlighted the poor quality of the studies performed in this discipline and the concomitant difficulty in analyzing the results [169]. Furthermore, given the allergenicity of the components of BV, cases of allergenic reactions and anaphylactic responses to BV treatment have also occurred [170]. In this perspective, other authors have emphasized that safety studies have not been developed on clinical application of BV and factors such as the incidence of adverse events, dose, frequency or form of administration have not been established or critically assessed in depth [3,168]. 

## 5. Future Perspectives and New Approaches 

As discussed above, despite the number of scientific articles and reviews on BV, its components and applications, a lot of work still must be done in this area. Even though BV components are extensively reported on literature, their mechanisms of action and metabolic pathways are barely known [3]. Moreover, as it was previously reviewed, despite the potential of BV (and especially, BV acupuncture) in traditional medicine for the treatment of musculoskeletal disorders, more trials are essential for the setting of specific administration protocols and safety assurance [168]. Considering this fact, three strategies might be followed up. Firstly, (1) some researchers explored the possibility of obtaining what they named “essential bee venom”. This substance consisted in the purified venom filtered for PLA2 and histamine, in order to minimize allergic reactions and adverse effects while maintaining similar anti-inflammatory activity [171]. Secondly, (2) as it has been reviewed, some individual compounds of BV are responsible for beneficial health effects and, furthermore, safer and more effective, as is the case with melittin [168,171]. However, the complexity of BV has entailed analytical difficulties in terms of identification, quantification and, in particular, standardization [172]. Hence, analytical tools have become indispensable for analyzing marker compounds, as it is the case of mass spectrometry (MS) coupled to different ionization sources such as electrospray ionization (ESI) or matrix-assisted laser desorption/ionization (MALDI). These techniques will allow one to increase the current knowledge of BV components and also to discover new compounds [172]. These two strategies, (1) and (2), are in theory designed to ameliorate the role of BV acupuncture for the treatment of specific diseases [168]. Nevertheless, BV could be applied not only alone but in combination with modern medicine and mediated by synergistic effects, enhancing its clinical and therapeutic applications [3]. At last, (3) new approaches have been developed, especially in drug delivery and carrying systems. Drug delivery refers to the specific routes that a drug or another specific substance follows in the organism, aimed at promoting the efficacy and safety of the carried compound and lowering the incidence of adverse effects [173]. Each active molecule has a specific therapeutic window for a certain compound; thus, it is important to study cellular and molecular targets of BV and figure out the main routes of action as well as the main target sites [3,173]. Moreover, given the protein nature of BV, oral administration is difficult, as digestive enzymes act over it and degrade it. Its relatively short plasma half-life and the problematic nature of determining a concrete dose has promoted the development of other alternatives, such as the combination of active peptides with polymers or nanoparticles [174]. For instance, a study investigated the interactions occurred between BV and the copolymer poly(dl-lactide-co-glycolide-b-ethyleneglycol-b-dl-lactide-co-glycolide) (PLGA–PEG–PLGA) and found that the release of BV was decreased and the hydrogel was poorly degraded, whereas the exerted biological activity was maintained [175]. Previously, other authors suggested that BV coated with calcium alginate gel beads and entrapped in liposomes could be an efficient carrying or delivery system for BV [174]. Other studies have assessed the efficacy of venom peptides such as melittin with lytic capacity that combined with pH-sensitive polymer micelles are able to provide selective and potential capacity for effective intracellular delivery of other peptides [176]. In 2018, Lee et al. demonstrated the efficacy of chitosan/alginate nanoparticles for encapsulating BV and their activity against porcine reproductive and respiratory syndrome virus. In their study, they found that nasal-derived nanoparticles were capable of inducing Th1 immune response and increasing the production of CD4+, T lymphocytes and Th memory cell populations, of the cytokines IFN-γ, IL-12 and the transcriptional factors (STAT4 and T-bet). Likewise, it produced a decrease in the T regulatory cells, cytokines IL-10 and TGF-β and the transcriptional factors STAT5 and Foxp3 [177]. The development and integration of these strategies is aimed at achieving a better comprehension of the mechanisms of action of BV and a safer use directed towards the development of pharmaceutical formulations [172].

## 6. Conclusions

BV is a complex mixture of substances which has been used in traditional medicine and extensively investigated due to their biological properties. Its composition is fundamentally formed by proteins and peptides, although other molecules are also present but in a low concentration. Among its components, melittin is the most abundant and studied compound of BV, followed by PLA2, an enzyme that is considered (together with histamine) the main allergenic substance of BV. Besides, further study is necessary, especially on minor components of BV. Regarding its biological properties, several of them have been reported although research focuses on anti-inflammatory and immunomodulatory effects. However, it is essential to further study the action mechanism of BV, by performing more experiments both in vitro and in vivo. The main applications of BV would be referred to therapeutic purposes because of its positive effects on some diseases such as musculoskeletal and neurodegenerative diseases. These days, efforts are directed towards the establishment of safer doses and practices combined with new tendencies such as innovative delivery systems to minimize adverse effects. Furthermore, research moves forward the complete understanding of the routes of BV components and assessing its therapeutic application.

## Figures and Tables

**Table 3:** Antimicrobial activity of biological compounds. Modified from [1] and [127].

Component	Organism	Effective Dose (µg/mL)	Component	Organism	Effective Dose (µg/mL)
BV	*Acinetobacter baumannii* BAA	MIC 30	Melittin	*Acinetobacter baumannii* BAA	MIC 30
BV	*Bacillus subtilis*	MIC 8	Melittin	*Candida krusei*	MIC 30
BV	*Candida albicans*	MIC 60	Melittin	*Candida krusei*	MIC 30
BV	*Candida krusei*	MIC 60	Melittin	*Escherichia coli*	MIC 30
BV	*Candida parapsilosis*	MIC 60	Melittin	*Streptococcus pyogenes*	MIC 10
BV	*Clindamycin-resistant P. acnes*	MIC 0.067	Melittin	*Staphylococcus aureus* Amme	MIC 6
BV	*Enterococcus casseliflavus*	MIC 10	Melittin	*Streptococcus agalactiae*	MIC 30
BV	*Escherichia coli*	MIC 60	Melittin	MRSA	MIC 10
BV	*Klebsiella pneumoniae*	MIC 30	Melittin	*Bacillus subtilis*	MIC 6
BV	*MRSA*	MIC 60	Melittin	*Klebsiella oxytoca*	MIC 60
BV	*Propionibacterium acnes*	MIC 0.086	Melittin	*Staphylococcus aureus* BAA	MIC 8
BV	*Shigella flexneri*	MIC 60	Melittin	*Staphylococcus aureus*	MIC 10
BV	*Staphylococcus aureus*	MIC 10	Melittin	*Staphylococcus saprophyticus*	MIC 10
BV	*Staphylococcus aureus* Amme	MIC 60	Melittin	*Staphylococcus aureus* Amme	MIC 6
BV	*Staphylococcus aureus* BAA	MIC 30	Melittin	*Candida candida*	MIC 9.961
BV	*Staphylococcus epidermidis*	MIC 0.104	Melittin	*Staphylococcus epidermidis*	MIC 10
BV	*Staphylococcus epidermidis*	MIC 60	Melittin	*Lactobacillus casei*	MIC 4
BV	*Staphylococcus saprophyticus*	MIC 10	Melittin	*Enterococcus faecalis*	MIC 6
BV	*Streptococcus agalactiae*	MIC 40	Melittin	*Candida krusei*	MIC 30
BV	*Streptococcus pyogenes*	MIC 0.121	Melittin	*Listeria monocytogenes*	MIC 12.5
BV	*Streptococcus thermophilus*	MIC 30	Melittin	*Escherichia coli*	MIC 56.92
PLA2	*Citrobacter freundii*	MBC 1000	Melittin	*Staphylococcus aureus*	MIC 8.5
PLA2	*Enterobacter clocae*	MBC 10000	Melittin	*Staphylococcus aureus amme*	MIC 6
PLA2	*Escherichia coli*	MBC 10000	Melittin	*Enterococcus casseliflavus*	MIC 8
PLA2	*Lactobacillus caser*	MBC 400	Melittin	*Enterococcus faecalis* VanB	MIC 50
PLA2	*Trypanosoma brucei*	MBC 1	Melittin	*Enterococcus faecalis*	MIC 30

Minimal inhibitory concentration (MIC), minimal bactericidal concentration (MBC), effective concentration (EC_50_), methicillin-resistant Staphylococcus aureus (MRSA), vancomycin-resistant enterococci (VRE).

## Abbreviations

*Generic*	
BV	Bee Venom
DW	Dry Weight
RA	Rheumatoid Arthritis
PD	Parkinson’s Disease
MS	Multiple Sclerosis
LF	Liver Fibrosis
CNS	Central Nervous System
AD	Alzheimer’s Disease
*Components*	
Aa	Amino acid
PLA2	Phospholipase A2
MCD	Mast Cell-Degranulating Peptide
NO	Nitric Oxide
bPLA2	PLA2 Derived From Bee
MDA	Malondialdehyde
ASA	Acetylsalicylic Acid
LPS	Lipopolysaccharides
DPIV	Dipeptidyl Peptidase IV
GST	Glutathione S-Transferase
GSH	Glutathione Content
GPx	Glutathione Peroxidase
SOD	Superoxide Dismutase
Kir	Inward Rectifier Potassium
*Cellular components*	
TLR	Toll-Like Receptors
DR	Death Receptor
CD	Cluster of Differentiation
TNF	Tumor Necrosis Factors
TAM	Tumor-Associated Macrophages
IL	Interleukin
NF-kB	Inhibitory effect of the nuclear factor kappa-B
*Antioxidant activity*	
TBARS	Thiobarbituric Acid Reactive Substances
TAS	Plasma Total Antioxidant Status
TOS	Total Oxidant Status
OSI	Oxidative Stress Index
FRAP	Ferric Reducing/Antioxidant Power
ABTS	2, 20-Azinobis 3-Ethylbenzothiazoline-6-Sulfonic Acid
TAC	Total Antioxidant Capacity
